# Correlation of surface-enhanced Raman scattering (SERS) with the surface density of gold nanoparticles: evaluation of the critical number of SERS tags for a detectable signal

**DOI:** 10.3762/bjnano.10.102

**Published:** 2019-05-10

**Authors:** Vincenzo Amendola

**Affiliations:** 1 Department of Chemical Sciences, University of Padova, Padova, Italy

**Keywords:** discrete dipole approximation (DDA), gold nanoparticles (AuNPs), nanotags, surface-enhanced Raman scattering (SERS), surface plasmon resonance (SPR)

## Abstract

The use of plasmonic nanotags based on the surface-enhanced Raman scattering (SERS) effect is highly promising for several applications in analytical chemistry, biotechnological assays and nanomedicine. To this end, a crucial parameter is the minimum number of SERS tags that allows for the collection of intense Raman signals under real operating conditions. Here, SERS Au nanotags (AuNTs) based on clustered gold nanoparticles are deposited on a substrate and analyzed in the same region using Raman spectroscopy and transmission electron microscopy. In this way, the Raman spectra and the surface density of the SERS tags are correlated directly, showing that 1 tag/µm^2^ is enough to generate an intense signal above the noise level at 633 nm with an excitation power of only 0.65 mW and an acquisition time of just 1 s with a 50× objective. The AuNT density can be even lower than 1 tag/µm^2^ when the acquisition time is extended to 10 s, but must be increased to 3 tags/µm^2^ when a 20× objective is employed under the same excitation conditions. In addition, in order to observe a linear response, it was found that 10 SERS AuNTs inside the probed area are required. These findings indicate that a better signal-to-noise ratio requires high-magnification optics, while linearity versus tag number can be improved by using low-magnification optics or a high tag density. In general the suitability of plasmonic SERS labels for ultrasensitive analytical and biomedical applications is evident.

## Introduction

In surface-enhanced Raman scattering (SERS), the Raman scattering cross-section of molecules adsorbed on the surface of plasmonic nanostructures is enormously increased compared to the same isolated molecules [1–5]. In particular, the SERS enhancement factor can reach values as high as 10^12^, which can be attributed to two phenomena, the local electric field enhancement due to the surface plasmon resonance of the metal nanostructure (electromagnetic enhancement) and the charge transfer between the molecule and the metal substrate (chemical enhancement) [6–8]. In addition, given the generally low Raman scattering cross-section of molecules, Raman signals are exceptionally intense when the SERS effect occurs simultaneously with the electronic resonance of the molecule at the excitation wavelength used for Raman spectroscopy, a condition called surface-enhanced resonant Raman scattering [9–12]. Resonant SERS allows the generation of Raman scattering signals as intense as that of fluorescent compounds and, in fact, can be exploited for Raman labelling [13–17]. A SERS tag is typically composed of a plasmonic nanostructure capable of large electromagnetic field enhancement, coated with organic molecules (Raman reporters) resonant at the probe wavelength, where the entire structure is embedded in a stabilizing matrix [13,16–19]. When selectivity is required, the surface of the Raman tag should expose a targeting function for binding the analyte [13,16–17]. When the analytes can be concentrated on a surface or in a well-defined volume, SERS tags allow for the collection of an intense Raman signal revealing the presence of the targeted molecule. Analyte accumulation on a surface may happen, for instance, through a sandwich configuration as in the well-established enzyme-linked immunosorbent assay (ELISA) tests, but the analyte may also be naturally immobilized on a surface, such as an antigen overexpressed in cancerous cell membrane [20–22]. SERS labels are also useful to probe the uptake of nanomaterial inside living cells [23–26].

There are many advantages connected to the use of Raman tags, mostly related to the intensity and narrow bandwidth of their Raman peaks. SERS labels can be even brighter than semiconductor quantum dots; for example, the high intensity allows for detection using only a single nanotag with an ordinary Raman spectrometer [27–28]. The narrowness of the Raman bands allows multiplexing analysis by associating Raman reporters with different, non-overlapping peaks, which serve as a spectroscopic fingerprint that can be associated with different analytes [18,29–30]. Additional favorable features of SERS tags are their photostability, namely the absence of blinking or bleaching, and the possibility to excite in the red or near infrared spectral range, where most matrixes and substrates have low fluorescence background [13,17].

Therefore, due to the increasing demand for ultrasensitive identification and quantification of specific analytes or substrates such as cancerous tissues, SERS tags are the subject of intense investigation and continuous performance improvement in analytical chemistry, nano-biotechnology and nanomedicine [13,15,21,31]. For all of these applications, the crucial parameter is the minimum number of SERS tags required for the detection of an intense signal in real operating conditions [13,18,32–33], which often employ an ordinary micro-Raman spectroscopy set up [34] or portable Raman spectrometer [35–37]. In recent years, this has fostered a number of studies aimed at quantifying SERS performance from plasmonic nanoparticles dispersed on a substrate [38], inside microcavities [39], or even while monitoring electrochemical reactions [40].

This work reports on the study of SERS tags obtained by laser ablation synthesis in liquid solution (LASiS) of gold (Au) nanoparticles, their coating with three different Raman reporters that are resonant at 633 nm, and their stabilization with a biocompatible and hydrophilic coating. Their performance was tested by correlating the Raman signal to the density of Au nanotags (AuNTs) per unit area, as obtained by transmission electron microscopy (TEM) analysis of the same area probed by Raman spectroscopy. In this way, the minimum number of AuNTs required to generate a detectable signal under ordinary experimental conditions can be identified. In addition, a threshold or minimum number of AuNTs within the probed area that can produce a linear response is identified, which can be obtained more easily with low-magnification optics when the surface density of the SERS tags is as low as a few units per micrometer square. These results contribute to the optimization of the experimental conditions for the use of SERS tags in analytical and biomedical analysis with high sensitivity.

## Experimental

### Synthesis of Au nanotags

AuNPs with an average diameter of 10 ± 5 nm and log-normal size distribution (see Figure S1 in Supporting Information File 1) were obtained by LASiS using an Nd:YAG laser (1064 nm, 9 ns, 50 Hz) focused to 8 J/cm^2^ with a 10 cm focus lens on a 99.9% pure gold plate placed at the bottom of a cell containing a 10^−4^ M NaCl solution in double distilled water [18–19,41]. Dye solutions of either hexacyanin 3 (HITC, perchlorate, Exciton), malachite green (MG, oxalate salt, Sigma-Aldrich) or malachite green isothiocyanate (MGITC, Invitrogen) were added to a 2 nM AuNP solution at a 1:100 volume ratio. After 30 min under mild stirring, an aqueous solution of thiolated polyethylene glycol (PEG, MW 5000, Sigma-Aldrich) was added to the AuNPs with a final concentration of 30 µM. After stirring for 14 h at room temperature, the mixtures were washed with deionized water four times by centrifugation at 3000 rcf for 10 min and finally filtered with hydrophilic 0.45 µm cellulose filters. The reproducibility of the procedure was successfully verified on two distinct batches of MG-labelled AuNTs dispersed in water (Figure S2 in Supporting Information File 1).

The samples for TEM analysis were obtained by mixing the AuNT solution (0.2 mg/mL in Au) 1:5 with a 10 mg/mL aqueous solution of polyvinyl alcohol (PVA, 200,000 *M*_w_, on average, from Fluka) and depositing one drop on a copper grid coated with a holey carbon film, according to a well-established procedure which serves to prevent particle agglomeration after drying the drop [42].

### Characterization

UV–visible spectroscopy was performed with a Varian Cary 5 spectrometer in 2 mm optical path length quartz cells. The AuNP concentration was estimated from UV–visible spectra and the application of Mie theory, as previously reported [43]. Transmission electron microscopy (TEM) was performed on an FEI Tecnai G2 12 instrument operating at 100 kV and equipped with a TVIPS CCD camera. The micro-Raman measurements were recorded with a 20× (NA 0.40, 64% coverage) or a 50× (NA 0.75, 100% coverage) Olympus objective on the micro-Raman instrument (CCD detector with 100 mm slits) on the TEM grids containing the AuNTs and using the 633 nm line of a He–Ne laser. The laser power at the entrance pupil of the microscope objective was 0.85 mW, corresponding to 0.65 mW at the output of the microscope objective (measured with a Scientec Vector calorimeter). The Raman signal was collected on an internal silicon chip which could account for small (less than 5%) intensity fluctuations of the Raman spectrometer and allowed for quantification of the noise intensity in the two measurement conditions. The acquisition time was fixed at 100 s.

### Numerical calculations

The local field, *E*_loc_, was calculated with the discrete dipole approximation (DDA) method using the software DDSCAT 7.1 and the related DDFIELD code [44–46]. A nanoaggregate of Au nanoparticles was created with same structure taken from a representative TEM picture of a real AuNT, and two different polarization directions were considered, namely parallel and perpendicular to the main axis of the nanoaggregate. For metal particles in the 2–200 nm size range, an error smaller than 10% is achieved using a number of dipoles at least of the order of 10^4^ and using an interdipole spacing much smaller than the wavelength of interest [44–45,47]. Therefore, in the present case, 4 × 10^5^ dipoles were used for the target, corresponding to an interdipole spacing of less than 0.5 nm.

## Results and Discussion

**Figure 1:**
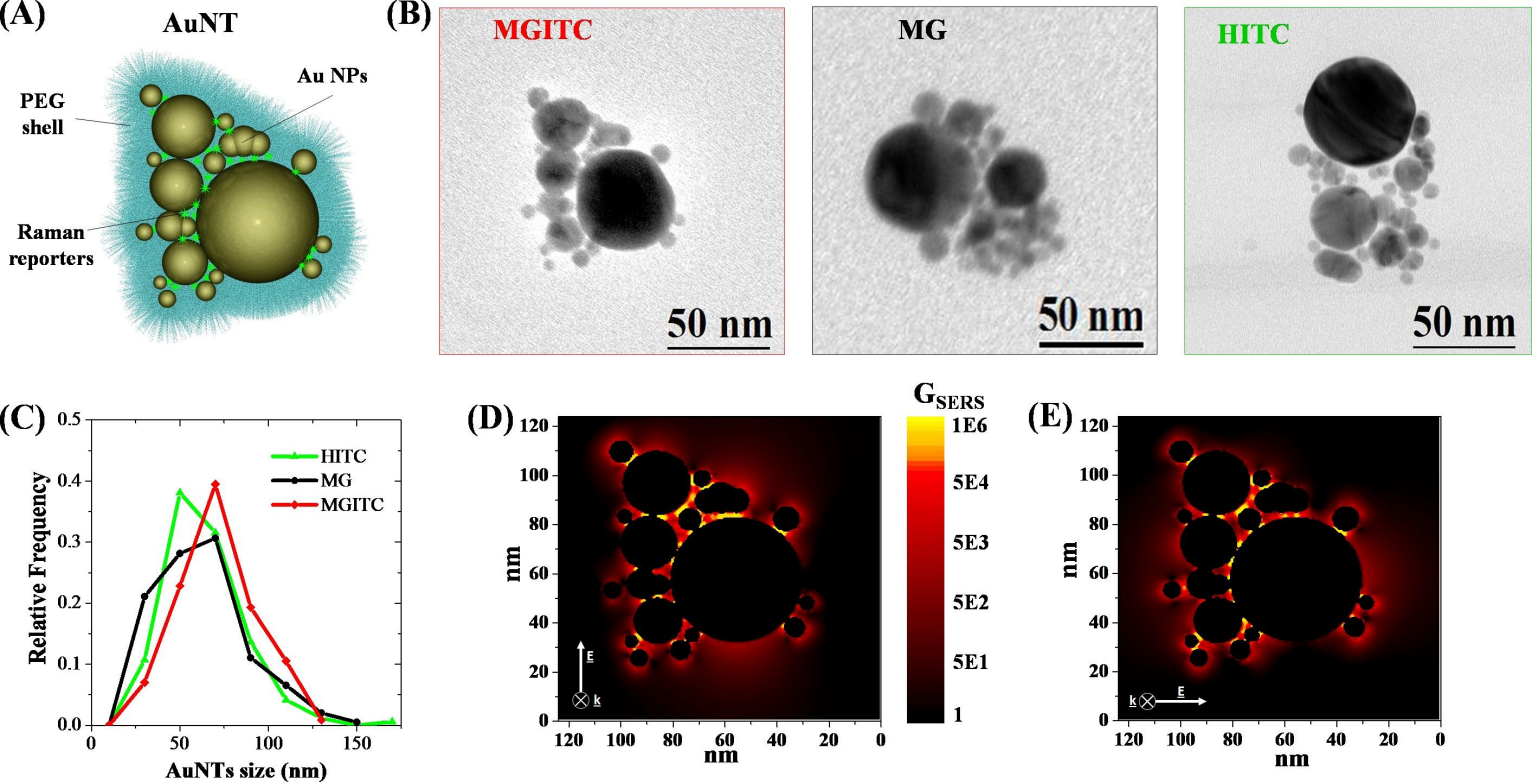
(A) Pictorial representation of the Au nanotag (AuNT), consisting of Au nanoparticles (NPs) aggregated by electrostatic interaction with cationic Raman reporters (either MG, MGITC or HITC), all coated with thiolated PEG. (B) Representative TEM images of MGITC, MG and HITC AuNTs. (C) Size histograms of HITC (green), MG (black) and MGITC (red) AuNTs, each one composed by several Au NPs. (D) *G*_SERS_ bidimensional map for a AuNT embedded in PVA under excitation at 633 nm with light polarized along the *y*-axis. (E) *G*_SERS_ bidimensional map for the same AuNT but with light polarized along the *x*-axis.

In this study, AuNTs consist of a cluster of Au nanoparticles aggregated in the presence of Raman active molecules, all coated with thiolated PEG (Figure 1A). The AuNTs assemble spontaneously because laser-generated Au nanoparticles are negatively charged and the selected Raman reporters are cationic molecules [19,48]. AuNTs containing MG, MGITC and HITC, examples of which are shown in Figure 1B, have a hierarchical structure with one or more large Au nanoparticles surrounded by smaller particles, all grouped in a nanoaggregate with size of the order of tens of nanometers. In particular, the size distribution of the nanoaggregates is similar among the three samples, as shown in Figure 1C, resulting in a comparable mean size of 63 ± 21 nm for HITC, 62 ± 25 nm for MG and 70 ± 22 nm for MGITC.

Importantly, the AuNTs show several junctions between plasmonic nanoparticles, which are well known sites of electromagnetic enhancement, as required for SERS [49–54]. This corresponds to a constellation of electromagnetic hot spots inside each nanoaggregate, where the local field enhancement is achieved in order to amplify the Raman signal of the adsorbed molecules by several orders of magnitude. This is further substantiated by numerical calculations of local field enhancement in a AuNT with structure reproducing the aggregate in Figure 1B. In particular, the SERS enhancement factor (*G*_SERS_) was obtained from the 4th power of the ratio between the local electric field, *E*_loc_, in the proximity to the surface of the metal nanostructure and the incident electric field, *E*_0_, from linearly polarized 633 nm electromagnetic radiation propagating in a medium with refractive index of PVA (*n* = 1.526) [6,55]. As shown in Figure 1D, *G*_SERS_ can reach values as high as 10^6^ and consistently between 10^5^–10^6^, depending on the hot spot considered.

Importantly, by changing the direction of polarization of incident light, the AuNTs always have several active hot spots, which reduces the number of “dark” tags in real operating conditions using polarized laser light sources, such as in ordinary Raman spectrometers [56]. This is demonstrated in Figure 1E, were the bidimensional map of *G*_SERS_ in the AuNT was calculated for a different polarization direction: *G*_SERS_ values span the same range as in Figure 1D, but the hot spots are mostly located at different points within the nanoaggregate.

**Figure 2:**
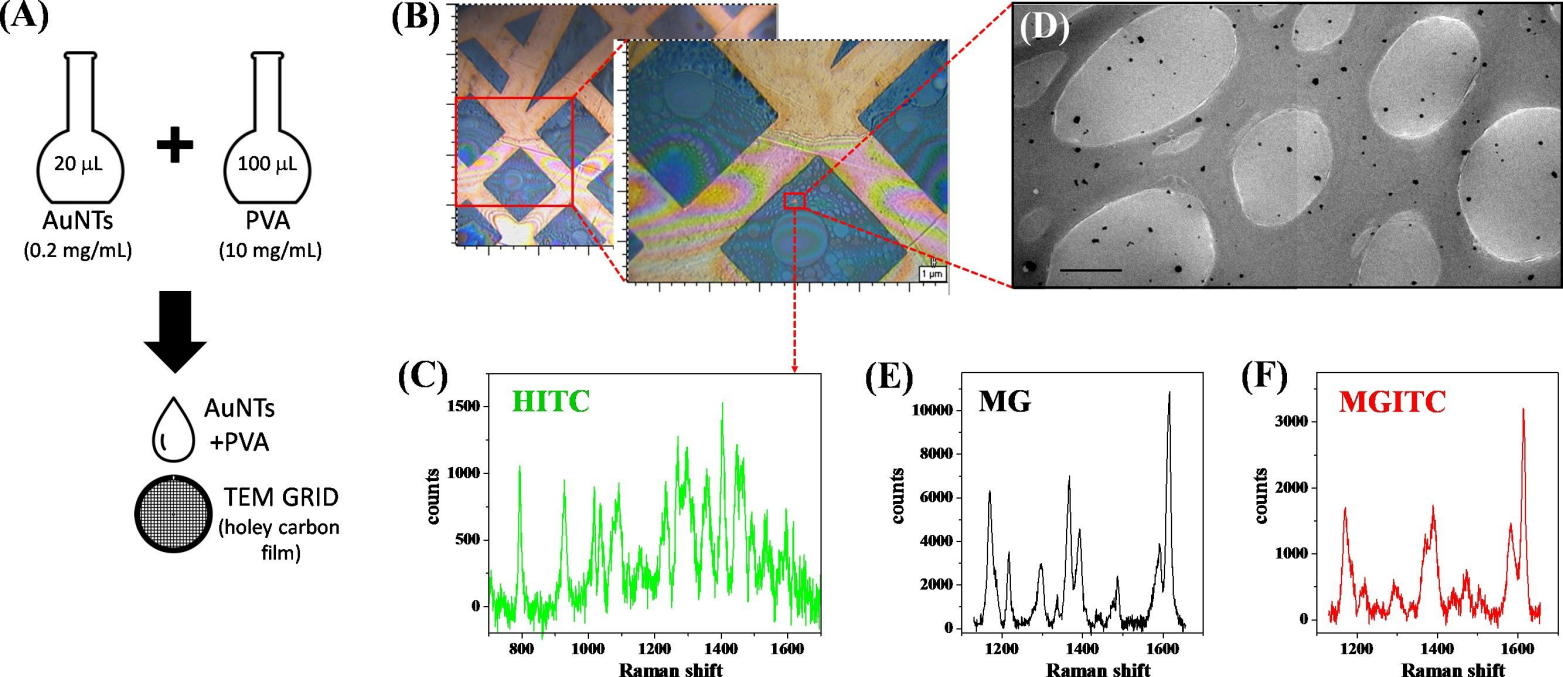
(A) Sketch of sample preparation for combined Raman and TEM analysis: 20 µL of an aqueous dispersion of AuNTs (0.2 mg/mL in Au) is mixed with 100 µL of an aqueous solution of PVA (10 mg/mL); after homogenization by ultrasonication, one drop of the mixture is deposited on a TEM grid coated with an holey carbon film. (B) 20× and 50× optical microscope images of the TEM grid coated with the PVA film embedding HITC–AuNTs. Some points of the grid are analyzed by Raman spectroscopy to collect the Raman spectra (C) and imaged with TEM (D) to obtain the surface density of the AuNTs. (E,F) Representative Raman spectra of MG and MGITC AuNTs collected with the same procedure.

To quantify the Raman scattering intensity versus concentration of nanoparticles, the three aqueous dispersions of AuNTs were mixed with a PVA solution and deposited on a TEM grid (Figure 2A). In this way, after evaporation of the liquid, the AuNTs remained dispersed in a PVA film with nanometric thickness (Figure 2B), according to a well-established procedure [42]. After sampling several points of each grid by Raman spectroscopy (see for instance Figure 2B,C), TEM images were collected in the same area (Figure 2D) in order to relate the surface density of AuNTs to their signal intensity.

The Raman spectra collected on the grids showed sharp signals clearly ascribable to the fingerprints of the three Raman reporters. The spectrum of HITC shows a rich progression of vibrational bands over the whole range from 700 to 1700 cm^−1^, with the most intense band located at 1410 cm^−1^ (Figure 2C). For MG, distinctive vibrational peaks are present at about 1200, 1400 and 1600 cm^−1^, the most intense of which is peaked at 1614 cm^−1^ (Figure 2E). MGITC, being the isothiocyanate derivative of MG, has a very similar vibrational fingerprint with the most prominent band also centered at 1614 cm^−1^ (Figure 2F).

**Figure 3:**
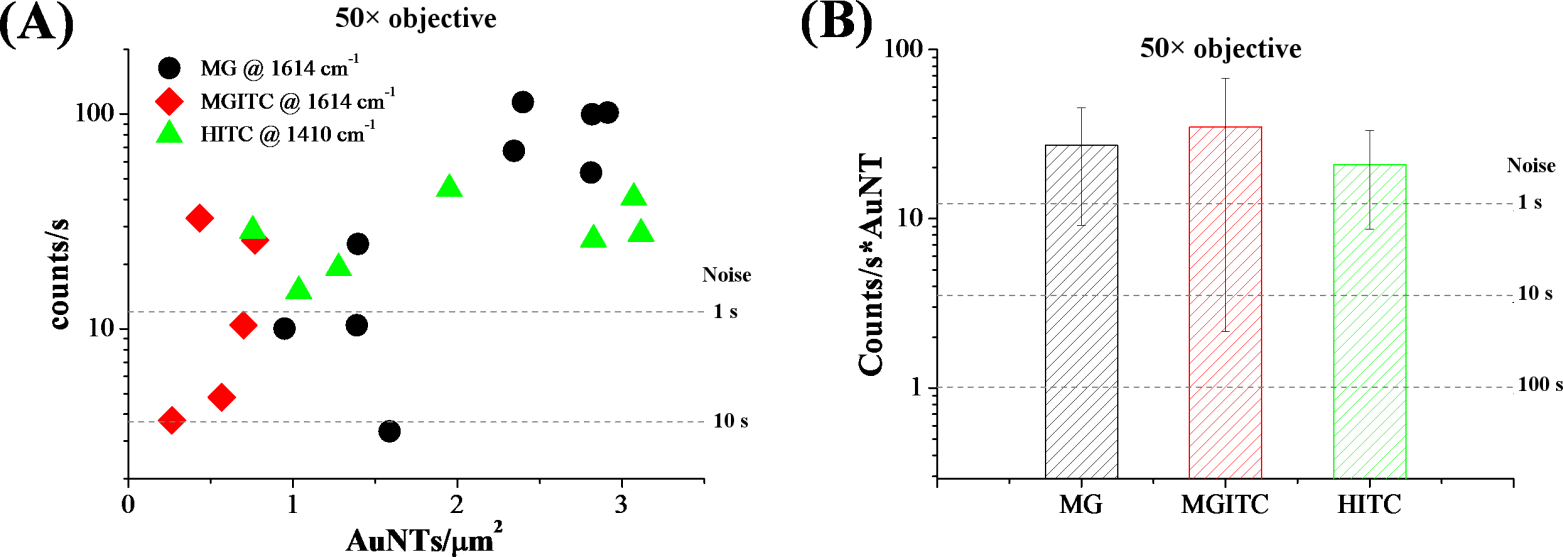
Results for Raman analysis with the 50× objective: (A) Counts per second versus AuNT surface density for MG (black circles), MGITC (red diamonds) and HITC (green triangles) labels. Each point in the graph corresponds to a different point on the TEM grid. (B) Mean value of the counts per second for single AuNT. Error bars represent the standard deviation. Dashed lines represent twice the noise level in the experimental conditions used and for various acquisition times.

In Figure 3A the results of Raman measurements performed with the 50× objective are reported. A clear signal is detectable above the noise level corresponding to only 1 s of acquisition time, even for a density of 2–3 AuNTs/µm^2^ in the MG and HITC labels and of less than 1 AuNT/µm^2^ in the MGITC case. For less than 1 AuNT/µm^2^, the signal intensity becomes generally comparable to the noise level at 1 s acquisition, but in most cases it is still higher than the noise level for 10 s of acquisition (the noise level scales as the square of the acquisition time) [57]. Considering the spot size of the laser beam on the sample, it is possible to evaluate the mean value and relative standard deviation of the counts per second from a single AuNT in the experimental conditions used (reported in Figure 3B), which is indicative of the average Raman scattering cross-section for a single label.

The signal from all of the three label types exceeds the noise level for 1 s of acquisition, meaning that, on average, one AuNT is enough to generate a well detectable signal even for such a short measurement time. From Figure 3B, it is also evident that the three labels exhibit comparable Raman intensity within the tolerance indicated by the error bars.

On the other hand, linearity is not observed in the plot of Figure 3A. Considering the random distribution of AuNTs on the TEM grids, this is attributable to the low surface density of nanolabels in combination with the small area probed with the 50× objective, which makes it highly probable that the effective number of labels probed with the laser spot changes by some units from one measurement to the other. Therefore, a higher AuNT density and/or larger spot size for Raman analysis is required to observe a linear correlation between counts per second and label density.

**Figure 4:**
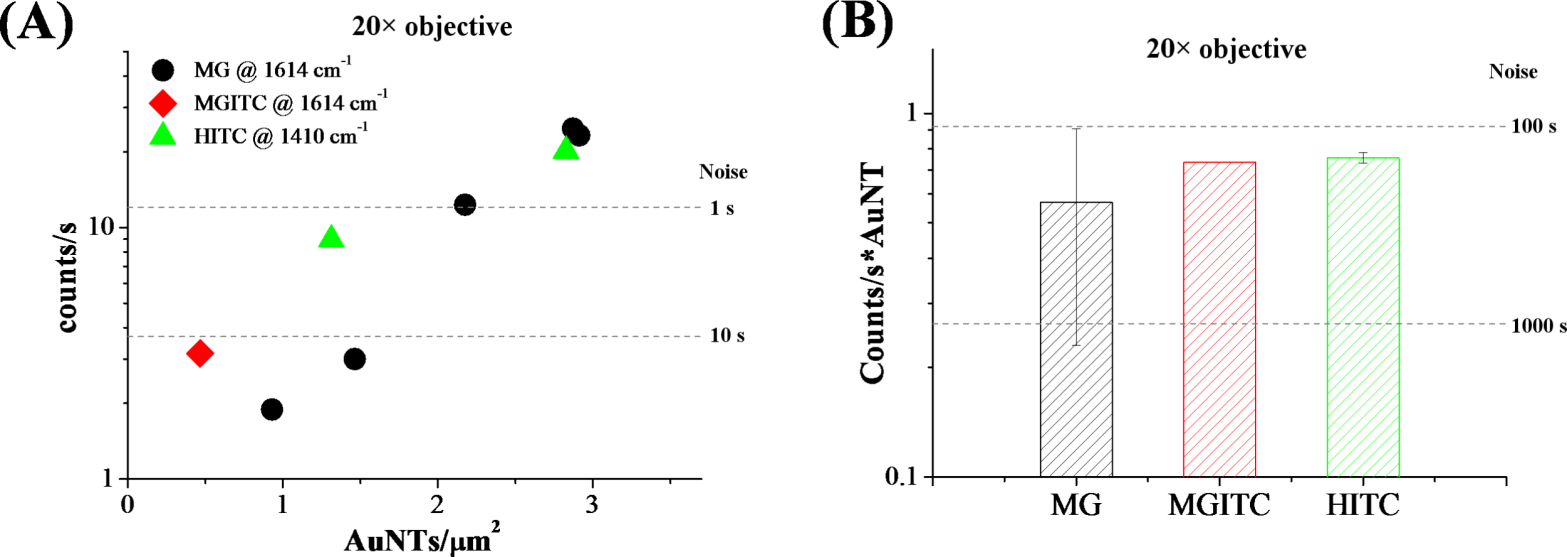
Results for Raman analysis with the 20× objective: (A) Counts per second versus AuNT surface density for MG (black circles), MGITC (red diamonds) and HITC (green triangles) labels. Each point in the graph corresponds to a different point on the TEM grid. (B) Mean value of the counts per second for a single AuNT. Error bars represent the standard deviation. Dashed lines represent twice the noise level in the experimental conditions used and for various acquisition times.

This hypothesis can be verified from the results of the Raman measurements performed with a 20× objective and the same excitation power, wavelength and exposure time as with the 50× objective, as reported in Figure 4A. In particular, in the case of the MG dataset, which includes a sufficiently large number of points, an appreciable correlation between AuNT density and counts per second is found. The MGITC and HITC points are not far from the MG ones, in agreement with the generally comparable brightness exhibited by the three types of AuNTs. The trend of the MGITC and HITC AuNTs also contributes to evidencing the expected growth of Raman signal with label density. This is noticeable since the AuNT preparation method, though simple and economic, does not allow high reproducibility of SERS response among all the single tags, as shown in Figure 3A.

The focused laser spot with the 20× objective is one order of magnitude larger than with the 50×, which corresponds to a lower laser intensity at a given input power, but also to a proportionally larger number of AuNTs inside the sampled area at a given of surface density. However, the numerical aperture and, thus, the solid angle of collection with the 20× objective is smaller than with the 50× objective, resulting in a signal decrease of more than one order of magnitude in our experimental conditions. Despite this, a density of 2–3 AuNTs/µm^2^ is still enough to obtain a well detectable signal with an acquisition time of only 1 s. However, the lower laser intensity and collection efficiency of the 20× objective is transformed into a lower mean value of counts per second from the single AuNT, that is now within the noise range for acquisition times of 100 and 1000 s (Figure 4B). This clearly indicates that objectives with a large numerical aperture provide a better response when the number of AuNTs per unit area is as low as 1 tag per µm^2^. It is worth stressing that acquisition times of hundreds of seconds are compatible with the AuNTs, which showed good photo and thermal stability also after laser exposure up to 1000 s with a 50× objective and 0.65 mW (see Figure S3 in Supporting Information File 1). However, it should be noted that a density of few SERS tags per micrometer square is much lower than that typically pursued in practical cases for both analytical and biomedical purposes [13,16–22].

The good performance of AuNTs is confirmed by the fact that no Raman signal was detected from the pure dyes without AuNTs under the same experimental conditions. Moreover, it is well known from literature that the utilization of Au NPs of the same size is associated with higher SERS enhancement factors [18], therefore suggesting that the performance of the AuNTs can be further improved by employing size-selected nanospheres.

## Conclusion

In this study, the performance of SERS labels based on Au NPs and organic dyes resonant at 633 nm was investigated by a combination of Raman and TEM analysis. The AuNTs were designed in order to support multiple electromagnetic hot spots for any polarization direction of the excitation beam.

The results highlight the appreciable intensity of the AuNTs, which allows a clear detection of the Raman signal above the noise threshold with a surface density of only 1 tag/µm^2^ when using an excitation power of only 0.65 mW at 633 nm with a 50× objective and acquisition times as short as 1 s. The tag density can be lowered even below 1 tag/µm^2^ if the acquisition time can be extended to 10 s, but it should be increased to 3 tags/µm^2^ with a 20× objective under the same excitation conditions. In case of an ultra-low AuNT density, a threshold of the total number of SERS tags inside the probed area is required to obtain a linear response, that is, on the order of 10 tags. Overall, these results suggest that a better signal-to-noise ratio requires optics with a high numerical aperture, while linearity versus tags number is improved by using low-magnification optics. However, it should be noted that the power density and tag density in this study are much lower than that typically achieved in real applications, further emphasizing the appreciable intensity of these AuNTs. Therefore, these results are useful for and contribute to the exploitation of AuNTs as ultrabright Raman tags in analytical chemistry, biotechnological assays and nanomedicine.

## Supporting Information

File 1 Size distribution of Au nanoparticles, UV–visible spectra of Au nanotags, Mie theory fit results, MG-AuNT reproducibility, and MG-AuNT photostability.
