# Supplementary material for: Correlation of surface-enhanced Raman scattering (SERS) with the surface density of gold nanoparticles: evaluation of the critical number of SERS tags for a detectable signal
Source: Beilstein J Nanotechnol. 2019 May 10;10:1016–23. doi: 10.3762/bjnano.10.102 (PMC6541332; doi:10.3762/bjnano.10.102)
Supplement: File 1 — Size distribution of Au nanoparticles, UV–visible spectra of Au nanotags, Mie theory fit results, MG-AuNT reproducibility, and MG-AuNT photostability. [file Beilstein_J_Nanotechnol-10-1016-s001.pdf]

BEILSTEIN JOURNAL OF NANOTECHNOLOGY

Supporting Information

for

Correlation of surface-enhanced Raman scattering (SERS) with the surface density of gold nanoparticles: evaluation of the critical number of SERS tags for a detectable signal

Vincenzo Amendola

Beilstein J. Nanotechnol. **2019,** *10,* 1016–1023. doi:10.3762/bjnano.10.102

Size distribution of Au nanoparticles, UV–visible spectra of Au nanotags, Mie theory fit results, MG-AuNT reproducibility, and MG-AuNT photostability

License and Terms: This is a supporting information file under the terms of the Creative Commons Attribution License (http://creativecommons.org/licenses/by/4.0). Please note that the reuse, redistribution and reproduction in particular requires that the authors and source are credited.
The license is subject to the *Beilstein Journal of Nanotechnology* terms and conditions: (https://www.beilstein-journals.org/bjnano)

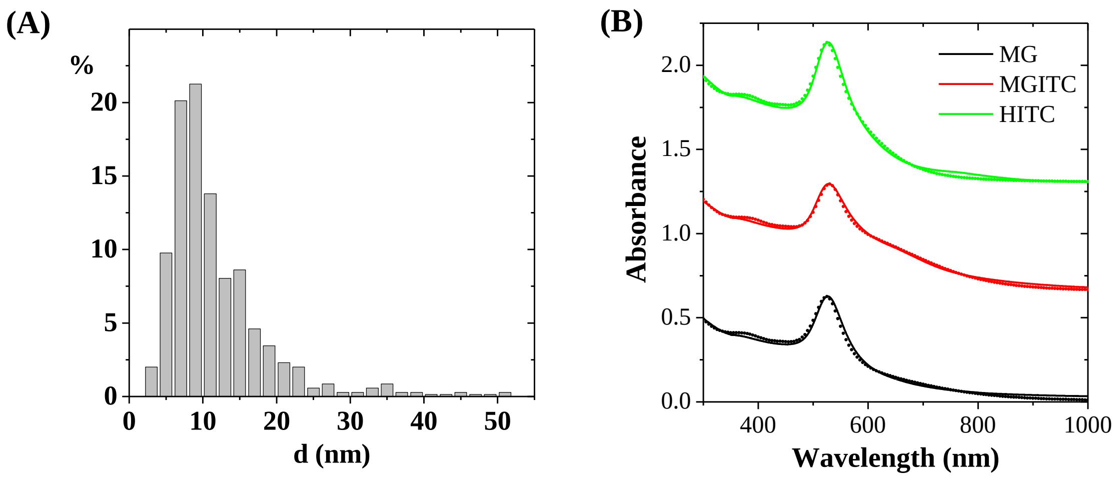

Figure S1: (A) Size distribution of Au NPs used for the realization of SERS tags, showing the typical lognormal structure obtained by laser ablation in liquid, with a peak at about 10 nm and a tail at larger size. (B) UV–vis absorption spectra (continuous line) and fit with the Mie theory (circles) of the MG, MGITC and HITC AuNTs dispersions in water. Fitting results are reported in Table S1. Spectra are vertically shifted for clarity of visualization. No changes to the spectra were observed after Raman analysis.

Table S1: Results of fits with Mie theory.

Sample	Au concentration
MG	0.19 mg/mL
MGITC	0.20 mg/mL
HITC	0.24 mg/mL

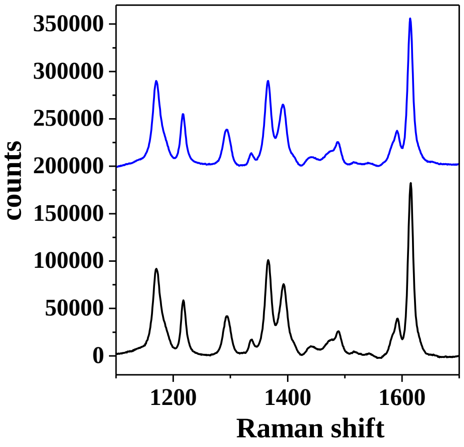

Figure S2: SERS spectra collected on two distinct batches of MG-AuNTs dispersed in aqueous solution, showing similar signal intensity. Spectra were collected with a 5× objective at 633 nm (13 mW) for 60 s.

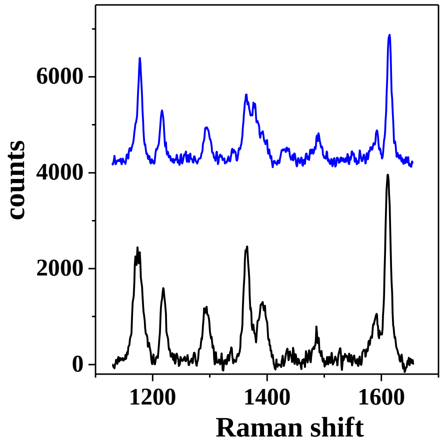

Figure S3: Stability versus time of the SERS spectra of MG-AuNTs deposed on the TEM grid. The sample was subjected to 633 nm laser exposure with a 50× objective and a power of 0.65 mW. Raman spectra were collected with an accumulation time of 100 s, before (black line) and after (blue line) 1000 s of continuous laser exposure.
